# A latent-class heteroskedastic hurdle trajectory model: patterns of adherence in obstructive sleep apnea patients on CPAP therapy

**DOI:** 10.1186/s12874-021-01407-6

**Published:** 2021-12-01

**Authors:** Niek G. P. Den Teuling, Edwin R. van den Heuvel, Mark S. Aloia, Steffen C. Pauws

**Affiliations:** 1 Dep. of Mathematics and Computer Science, Eindhoven University of Technology, Eindhoven, The Netherlands; 2 Philips Research, Eindhoven, The Netherlands; 3 Department of Medicine, National Jewish Health, Denver, CO USA; 4 Philips Respironics, Monroeville, PA USA; 5 Department Communication and Cognition, Tilburg University, Tilburg, The Netherlands

**Keywords:** Obstructive sleep apnea, CPAP therapy, Treatment adherence, Latent-class trajectory modeling, Multilevel mixture modeling, Hurdle modeling, Heteroskedastic modeling, Intensive longitudinal data

## Abstract

**Background:**

Sleep apnea patients on CPAP therapy exhibit differences in how they adhere to the therapy. Previous studies have demonstrated the benefit of describing adherence in terms of discernible longitudinal patterns. However, these analyses have been done on a limited number of patients, and did not properly represent the temporal characteristics and heterogeneity of adherence.

**Methods:**

We illustrate the potential of identifying patterns of adherence with a latent-class heteroskedastic hurdle trajectory approach using generalized additive modeling. The model represents the adherence trajectories on three aspects over time: the daily hurdle of using the therapy, the daily time spent on therapy, and the day-to-day variability. The combination of these three characteristics has not been studied before.

**Results:**

Applying the proposed model to a dataset of 10,000 patients in their first three months of therapy resulted in nine adherence groups, among which 49% of patients exhibited a change in adherence over time. The identified group trajectories revealed a non-linear association between the change in the daily hurdle of using the therapy, and the average time on therapy. The largest difference between groups was observed in the patient motivation score. The adherence patterns were also associated with different levels of high residual AHI, and day-to-day variability in leakage.

**Conclusion:**

The inclusion of the hurdle model and the heteroskedastic model into the mixture model enabled the discovery of additional adherence patterns, and a more descriptive representation of patient behavior over time. Therapy adherence was mostly affected by a lack of attempts over time, suggesting that encouraging these patients to attempt therapy on a daily basis, irrespective of the number of hours used, could drive adherence. We believe the methodology is applicable to other domains of therapy or medication adherence.

## Background

For clinical efficacy, patients need to adhere to the prescribed medical treatment. The degree to which patients are successful in adhering to their treatment depends on the condition, dosing frequency, treatment duration, and many other factors [1]. Another aspect of interest is the change in adherence over time, of which an improved understanding can contribute to the early prediction of non-adherence and help in selecting the appropriate intervention. Patient adherence can either be modeled in terms of a common time trend from which patients exhibit random structural deviations, or as a stratified analysis comprising subgroups of patients with specific longitudinal patterns.

In this work we explore the longitudinal therapy adherence patterns that obstructive sleep apnea (OSA) patients exhibit during their first three months of continuous positive airway pressure (CPAP) therapy. Identifying common patterns of adherence provides population-level insights on how patients typically use the therapy. Moreover, it may guide new interventions for targeting the specific adherence behaviors, or help durable medical equipment providers with substantiating their reimbursement claims.

OSA is a chronic disorder involving frequent pauses in breathing during sleep. The disorder is common in the adult population, with the prevalence ranging from 9% to 38% [2], and increasing with age. The apneas in OSA arise from a collapse of tissue in the airway during sleep. The severity of the condition is typically measured in terms of the number of breathing disturbances per hour of sleep, referred to as the apnea-hypopnea index (AHI), where in severe cases of OSA these disturbances occur over 30 times per hour of sleep. Consequently, excessive daytime sleepiness, reduced quality of life, and increased risk of cardiovascular disease are among the side effects associated with OSA if left untreated [3].

CPAP is considered to be the first-line therapy for treating OSA. However, in order for the treatment to be effective, patients need to use it daily. The benefits of CPAP (e.g., reduced daytime sleepiness) can diminish after as early as one omitted day [4]. Furthermore, the dose-response relation between hours of usage and daytime sleepiness has been found to be linear, showing improved outcomes with up to 7 hours of usage per day [5]. The level of adherence to the therapy is quantified in terms of the daily number of hours the treatment was used.

While the majority of patients (66%) succeed in adjusting to CPAP therapy, others fail to start, give up early, or abandon the therapy within a couple of weeks or months [6]. Moreover, the consistency in the number of hours used varies between patients. On some days, patients do not initiate therapy, these days are referred to as intermittent days or non-attempts. The complexity of adherence is evident from the numerous factors that have been identified to be indicative of future CPAP adherence to some degree. This includes demographic factors such as age, sex, BMI, and socioeconomic class [7], and equipment-related factors such as the device type (e.g., continuous or automatic PAP), device features (e.g., heated humidification), and therapy-related side effects, e.g., mask discomfort, leakage, or skin abrasion [8]. Moreover, psychological factors have been identified [7], for example the knowledge of patients about the therapy, the belief in ability to control one’s health, the perceived risk and health benefit of the therapy, and motivation. In addition to the individual factors, external factors such as family, physician, health care professionals and facility all play a role in adherence [7].

Earlier studies have handled the heterogeneity of adherence by stratifying the patients on well-defined criteria. An example of this is found in the study by Weaver et al. [9], in which they observed a bimodal distribution for CPAP attempt consistency, with approximately half of the patients being highly consistent (over 90% attempted days). Wohlgemuth et al. [10] stratified patients based on the percentage of nights of usage, nights of usage above 4 hours, average nightly usage, and other factors. For each of these factors, the average was computed over the therapy duration, resulting in a cross-sectional cluster analysis. Using latent class analysis, they identified groups of non-adherers, attempters and adherers.

Other studies have explored how adherence changes over time across patients, with a focus on the daily time spent on therapy. Aloia et al. [11] investigated first-year CPAP therapy adherence among 71 patients in detail by visualizing individual daily time on therapy and manually grouping similar trajectories on time series characteristics (intercept, variance, slope, autocorrelation, and length). They found seven patterns of adherence. However, a limitation of their approach is that a manual evaluation is infeasible for a large number of patients. Babbin et al. [12] performed a similar time series analysis on 161 patients over 180 days, but they used an automated approach for clustering the adherence trajectories. The trajectories were classified into four clusters using agglomerative hierarchical clustering of the daily time on therapy as independent variables. They identified significant differences between groups on patient characteristics in a post-hoc analysis. Wang et al. [13] applied *k*-means among 76 patients, identifying three adherence patterns over the first 12 weeks of CPAP therapy. Moreover they showed that patients belonging to the cluster with poor adherence could be distinguished reliably from the other clusters at baseline.

Overall, the above mentioned studies have yielded varying adherence patterns of interest over different ranges of time. However, these analyses involved fewer than 250 patients, which puts an upper bound on the number of groups that can reliably be detected, and limits the power of the post-hoc group comparison. With respect to modeling the temporal aspect of adherence, the studies demonstrate the added value of describing adherence in terms of the attempts made and the mean level of usage, as well as the day-to-day variability in time on therapy.

We represent the adherence over time by combining the different approaches taken in previous studies. We model the daily time on therapy using latent-class distributional regression with time as a continuous covariate. Here, the daily patient usage is modeled as a two-stage process, where the daily action of initiating therapy is modeled over time as a hurdle that patients must pass before the time on therapy is modeled. Moreover, we model how the expected mean and variability of time on therapy changes over time. To the best of our knowledge, such an approach has not yet been used for modeling therapy adherence in patients (with sleep-disordered breathing). Hurdle modeling is typically used in areas involving count data, such as economics, epidemiology, healthcare utilization, and ecology. We will identify patterns of adherence in the therapy data on these three aspects by estimating a latent-class hurdle trajectory model, using a generalized additive modeling (GAMLSS) approach [14]. Furthermore, we compare several CPAP therapy-related external variables between groups, following a three-step approach [15, 16].

### Data

In the present study we analyze retrospective data collected from patients in the United States who are on CPAP therapy, and registered for and made use of the DreamMapper application made available by Philips Respironics. The DreamMapper application is available on mobile and the web, with the purpose of supporting patients in their first months of PAP therapy [17], and is free to use. Patients can connect their CPAP device or manually upload their CPAP device data to the DreamMapper application in order to gain insights into their therapy. We obtained CPAP device data from 37,235 patients who have manually uploaded data via Bluetooth or SD card to the DreamMapper application during their first 90 days of therapy, and have consented to the use of their uploaded data for research. From the available dataset, we selected a random sample of 10,000 patients to conduct the regression analyses^1^.

Patients included in the obtained dataset all meet the following criteria: Firstly, the patients started therapy and manually uploaded data between May 2017 and April 2018. Secondly, the patients started within a week of their DreamMapper registration date with CPAP therapy, and were first-time users of the therapy. This was determined by the absence of other user accounts created by them in potentially previous times. Lastly, only patients who have uploaded therapy data beyond their first 90 days of therapy were included. This ensures that they have been on therapy for at least 90 days, regardless of the number of days the therapy was used.

It is important to note that due to the 90-day therapy requirement, the typical level of adherence in our data is higher than what would be expected from a more general patient population. We focus on patients having been on therapy for at least 90 days for two reasons. Firstly, the lack of information on the reason for the data flow stopping means that we could not distinguish between patients who abandoned therapy and those who stopped uploading data but continued their therapy. Secondly, clustering trajectories while including patients of shorter therapy durations confounds the patterns of adherent patients with those who abandoned therapy. Considering that the interpretation and possible applications are different for these two cases, it is preferable to analyze and model the cases separately. Furthermore, this simplifies the required model for our analysis, as we do not need to account for censoring or different drop-out durations.

The available data per patient consists of daily aggregated CPAP device data, and a motivation assessment filled in on a voluntary basis. Patient demographics and other relevant baseline information such as the pretreatment AHI are not available for analysis. In addition to the daily amount of time patients were on therapy (the mask-on time, in seconds), as recorded by the CPAP device, we will compare the average residual AHI, average leakage, and pressure settings to identify differences between the identified groups. The motivation assessment is solicited at the very beginning of therapy, during onboarding. Patients are asked to rate their motivation to treat their sleep apnea condition on a scale from 1 to 10, where a 10 represents the highest possible level of motivation. A total of 2,973,759 observation days are available. Missing device data on intermittent days of therapy is assumed to be due to no attempt being made to use the therapy, as technical errors are deemed to be rare.

We computed the summary statistics on the complete dataset. On days during which patients used the therapy, the time on therapy is approximately normally distributed with a mean of 6.7 hours (SE 0.003) and a standard deviation of 2.1 hours (SE 0.001). The distribution is slightly left-skewed, with a skewness of -0.40 (SE 0.001). Remarkably, the average usage is considerably higher than the estimate of 5.8 hours by Hardy et al. [17] for patients who use the DreamMapper application in their first 90 days of therapy, which we suspect can be attributed to our exclusion of patients that stopped engaging with the app or their therapy before day 90.

**Fig. 1:**
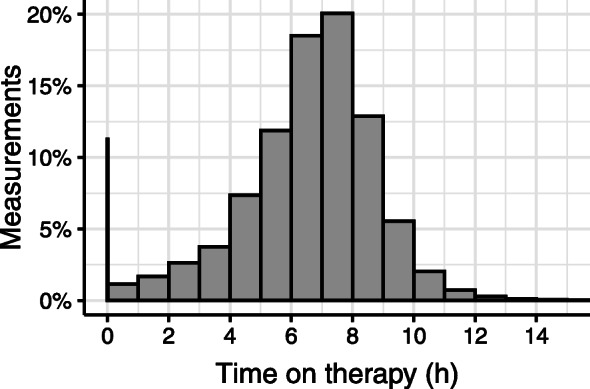
Distribution of time on therapy, with intermittent days represented by zero hours

On average, patients did not use the therapy on 11.3% of days. In order to correctly model adherence over time, we therefore include these intermittent days as observations with zero hours on therapy. However, this leads to a response variable with an excess of zeroes. The overall distribution of time on therapy is shown in Fig. 1, where intermittent days are represented by the vertical black line at zero.

The time on therapy ranges from 0 to 23 hours, but measurements exceeding 15 hours (0.05%) were removed because these relatively extreme values were considered to be unreliable measurements of the actual usage, and could affect the model estimation. In order to improve the robustness of the post-hoc analysis, extreme values from other covariates were removed for the same reason, using conservative thresholds based on the lower and upper 0.01% of values. In total, fewer than 1% of observations are affected by these processing steps.

## Methods

### Hurdle model

The excess zeroes that are present in the data cannot be ignored. In count data, this is typically addressed using a zero-inflated model [18], which models the increased probability of observing zeroes, in addition to the zeroes expected from the response distribution, e.g., a Poisson distribution. However, in the present study, the counts of the number of seconds on therapy are more closely represented by a normal distribution with a strictly positive domain (e.g., the log-normal or truncated normal distribution). Moreover, in this context, zeroes have only one interpretation, namely that of the therapy not being initiated on a given day. If we regard initiating treatment as a hurdle that patients need to overcome daily, we can model the initiation of therapy as a two-step process. This approach is referred to as hurdle modeling, and is generally applicable when a response variable is conditional on the occurrence of an event, with distinct values.

A hurdle model comprises a finite mixture of a point mass at zero, and a distribution with positive domain. Excess zeroes in a count variable can arise from a significant hurdle or a factor preventing the event from happening, but can also happen when the time available for counting the events is too short relative to the frequency of the event occurring. Lee et al. [19] investigated the risk of miscarriage in women with sleep-disordered breathing using truncated Poisson hurdle regression. Hurdle modeling is not restricted to count data, as it can be applied with any distribution that does not contain the hurdle response value (e.g., a truncated distribution). Saberi et al. [20] investigated the percentage of HIV medication non-adherence using a Gamma hurdle model.

Let $\phantom {\dot {i}\!}\mathbf {y}_{i}=\left \{ y_{i,1},y_{i,2},...,y_{i,J_{i}}\right \} $ denote the adherence trajectory of patient *i*∈*I* consisting of *J*_*i*_=90 observations, for any patient from the set of available patients *I*. Here, *y*_*i,j*_ denotes the time on therapy of patient *i* on the *j*th measurement at time *t*_*j*_. The daily hurdle of initiating therapy can be modeled with a Bernoulli process with probability

1 $$ \Pr\left(H_{i,j}=h_{i,j}\right)=\left\{\begin{array}{ll} \nu_{j} & h_{i,j}=0\\ 1-\nu_{j} & h_{i,j}=1 \end{array}\right.,  $$

where *h*_*i,j*_∈{0,1} denotes whether the hurdle is overcome for patient *i* at time *t*_*j*_, and *ν*_*j*_∈(0,1) represents the probability of failing to pass the hurdle at time *t*_*j*_.

**Truncated normal hurdle model** With the exception of models involving two-sided truncation, as seen in double hurdle modeling, examples of left-sided truncated normal distributions are few in number. Cragg [21] first proposed a truncated normal hurdle model for modeling the consumer demand of durable goods, to account for periods of time during which no purchases of goods were made. For observations where the hurdle is passed (i.e., *h*_*i,j*_=1), we assume the time on therapy *y*_*i,j*_ to be normally distributed with strictly positive values. The probability density function (PDF) is given by

2 $$ f_{\mathrm{N}}(y;\mu,\sigma)=\frac{\phi\left(\tfrac{(y-\mu)}{\sigma}\right)}{1-\Phi\left(\tfrac{-\mu}{\sigma}\right)}\quad y>0  $$

and zero otherwise, where *ϕ*(·) is the standard normal PDF, *Φ*(·) is the standard normal CDF, and *μ* and *σ* are the mean and standard deviation of the non-truncated normal distribution. If *X* has a normal distribution, the moments of the truncated normal distribution are then given by [22]

3 $$\begin{array}{*{20}l} {}\mathbf{E}(X|X >0)&= \mu+\sigma\frac{\phi\left(\tfrac{-\mu}{\sigma}\right)}{1-\Phi\left(\tfrac{-\mu}{\sigma}\right)},\\ {}\mathbf{E}\left(X^{2}|X >0\right)&= \mu^{2}+2\sigma\mu\frac{\phi\left(\tfrac{-\mu}{\sigma}\right)}{1-\Phi\left(\tfrac{-\mu}{\sigma}\right)}\\&\quad+\sigma^{2}\left[\frac{\tfrac{-\mu}{\sigma}\phi\left(\tfrac{-\mu}{\sigma}\right)}{1-\Phi\left(\tfrac{-\mu}{\sigma}\right)}+1\right], \end{array} $$

4 $$\begin{array}{*{20}l} {}\mathbf{E}\left(X^{3}|X > 0\right)&= \mu^{3}+\left[3\sigma\mu^{2}-\mu\sigma^{2}+\sigma^{3}\right]\frac{\phi\left(\tfrac{-\mu}{\sigma}\right)}{1-\Phi\left(\tfrac{-\mu}{\sigma}\right)}\\&\quad+ 3\sigma^{2}\mu\left[\frac{\tfrac{-\mu}{\sigma}\phi\left(\tfrac{-\mu}{\sigma}\right)}{1-\Phi\left(\tfrac{-\mu}{\sigma}\right)}+1\right]. \end{array} $$

The time on therapy for patient *i* at time *t*_*j*_ is distributed as

5 $$ \Pr\left(y_{i,j}\leq y\right)=\nu_{j}+(1-\nu_{j})F_{N}(y_{i,j},\mu_{j},\sigma_{j})  $$

with *y*∈**R** and *F*_*N*_ the truncated normal distribution function.

The truncated normal hurdle distribution described here, denoted by TNH, is represented by three parameters. We allow each of the parameters to change over time. As such, each patient *i* at the *j*th observation at time *t*_*j*_ is represented by the probability *ν*_*j*_ of failing to pass the hurdle, the expected conditional mean *μ*_*j*_ and standard deviation *σ*_*j*_. In a single-group analysis the hurdle and conditional time on therapy essentially operate on disjoint data, and therefore can be estimated separately, using logistic regression to model the hurdle, and truncated normal regression for the conditional time on therapy. However, this does not hold when the terms across the models are assumed to be correlated, or when a mixture of hurdle models is being estimated.

### Generalized additive modeling for location, scale and shape

GAMLSS is a method for modeling a numerical univariate response variable in terms of a general parametric distribution. Whereas generalized additive modeling (GAM) and generalized linear modeling (GLM) can only handle exponential family distributions and assume a variance as a function of the mean with a constant scaling factor [23, 24], GAMLSS can describe parametric response distributions by their mean (i.e., location *μ*), variance (i.e., scale *σ*) and shape (e.g., skewness and kurtosis) in terms of linear predictors and additive functions. Furthermore, through the inclusion of a distributional parameter for the excess zeros, hurdle and zero-inflated distributions can be handled.

GAMLSS was proposed by Rigby & Stasinopoulos [14, 25], and developed into a framework implemented in various packages in R [26, 27]. To describe our model in terms of GAM, let $\phantom {\dot {i}\!}\boldsymbol {y}_{i}^{\top }=\left (y_{i,1},\ldots,y_{i,J_{i}}\right)$ denote the longitudinal measurements of a patient *i*∈*I* among the sets of patients *I*, with *y*_*i,j*_∼TNH(*μ*_*i,j*_,*σ*_*i,j*_,*ν*_*i,j*_). Each of the distributional parameters can be described by a linear model, describing the $J=\sum _{i\in I}J_{i}$ observations across all patients. The PDF of the complete model is given by *f*_*Y*_(*y*_*i,j*_;*μ*_*i,j*_,*σ*_*i,j*_,*ν*_*i,j*_). For brevity, the predictor vector of length *J* for the *k*th distribution parameter is denoted by ***d***_*k*_, with ***d***_1_=***μ***,***d***_2_=***σ***, and ***d***_3_=***ν***. The general random effects GAMLSS model [28] for the *k*th distributional parameter is given by

6 $$ g_{k}\left(\boldsymbol{d}_{k}\right)=\boldsymbol{X}_{k}\boldsymbol{\beta}_{k}+\sum_{m=1}^{M_{k}}\boldsymbol{Z}_{k,m}\boldsymbol{\gamma}_{k,m},  $$

where *g*_*k*_(·) denotes the monotonic link function for the respective distribution parameter. The linear additive terms of the model are represented by a *J*×*L*_*k*_ design matrix denoted by ***X***_*k*_ for *L*_*k*_ fixed effects, with coefficients $\phantom {\dot {i}\!}\boldsymbol {\beta }_{k}^{\top }=\left (\beta _{k,1},\ldots,\beta _{k,L_{k}}\right)$. The *J*×*Q*_*k,m*_ design matrix ***Z***_*k,m*_ models the random effects with ***γ***_*k,m*_ as a vector of *Q*_*k,m*_ random variables. These random effects also allow for (penalized) smoothing as a function of an explanatory variable, e.g., cubic splines, P-splines, and fractional polynomials. An advantage of GAMLSS is that the random effects can be included in any of the distributional parameters, although this comes at the cost of increased computational complexity.

We limit the model complexity by only representing each distributional parameter using a linear parametric representation. In addition, the hierarchical nature of the longitudinal data needs to be taken into account. Patients have different levels of expected usage, variance, and attempts, arising from factors such as sleep schedule, quality of sleep, and tolerance to the therapy. We can account for these patient-specific differences by partitioning the random effects design matrix into patient-specific matrices. We only consider the case of *M*_*k*_=1 (i.e., a random intercept model for each distributional parameter), so we therefore omit the *m* subscript from the notation hereafter. The patient-specific random effects design matrix ***Z***_*k,i*_ of order *J*_*i*_×*Q*_*k*_ are concatenated to yield $\boldsymbol {Z}_{k}^{\top }=\left [\boldsymbol {Z}_{k,1}^{\top }\left |\boldsymbol {Z}_{k,2}^{\top }\right |\cdots \left |\boldsymbol {Z}_{k,|I|}^{\top }\right.\right ]$ [28]. The random effects vector is denoted by $\phantom {\dot {i}\!}\boldsymbol {\gamma }_{k,i}=(\gamma _{k,1,i},\ldots,\gamma _{k,Q_{k},i})$, with ***γ***_*k,i*_∼*N*(0,*Σ*_*k*_) for each of the distribution parameters, where *Σ*_*k*_ is the variance-covariance matrix for the random effects of the respective distribution parameter.

Although we observe a marginally better fit using smoothing functions of time, modeling change using linear additive terms is preferred in this analysis for its lower complexity, and greatly reducing computation time. We therefore model each of the distributional parameters using a second-order polynomial dependent on time. The identity link function suffices for the mean *μ*_*i,j*_, whereas a log link is used for the variance *σ*_*i,j*_ in order to ensure positive values. The hurdle probability *ν*_*i,j*_ is modeled using logistic regression by assuming a logit link $g_{3}(\nu _{i,j})=\log \left (\frac {\nu _{i,j}}{1-\nu _{i,j}}\right)$. Accordingly, the random effects model is given by

7 $$\begin{array}{*{20}l} \mu_{i,j} & =\beta_{1,0}+\beta_{1,1}t_{i,j}+\beta_{1,2}t_{i,j}^{2}+\boldsymbol{Z}_{1,i,j}\boldsymbol{\gamma}_{1,i}, \\ \log\sigma_{i,j} & =\beta_{2,0}+\beta_{2,1}t_{i,j}+\beta_{2,2}t_{i,j}^{2}+\boldsymbol{Z}_{2,i,j}\boldsymbol{\gamma}_{2,i}, \\ \log\frac{\nu_{i,j}}{1-\nu_{i,j}} & =\beta_{3,0}+\beta_{3,1}t_{i,j}+\beta_{3,2}t_{i,j}^{2}+\boldsymbol{Z}_{3,i,j}\boldsymbol{\gamma}_{3,i}. \end{array} $$

We will use this model to compare against the mixture model described in the next section.

#### Latent-class modeling

The findings from previous studies on CPAP adherence suggest a complex, non-normal distribution of adherence patterns [11, 12]. We therefore opt for a non-parametric approach to modeling the heterogeneity, by describing the patient-specific deviations from the population mean in terms of a finite number of structural deviations. In a cross-sectional data context, this approach is commonly referred to as finite mixture modeling [29]. This has the added benefit of accounting for the (possibly non-linear) relationship between the distributional parameters through the different clusters. In particular, an association can be expected between the attempt probability and the mean level of usage.

Growth mixture modeling (GMM) is an approach to modeling longitudinal change (i.e., a growth curve), accounting for patient heterogeneity by assuming each patient belongs to one of several unobserved (i.e., latent) classes [30–32]. The class models include patient-specific random effects, therefore the approach essentially assumes the heterogeneous data to consists of a set of heterogeneous subgroups.

The appeal of allowing for patient-specific deviations within the latent classes is that it enables an emphasis on the change of adherence over time as opposed to the expected average time on therapy. Without a random intercept, most of the group trajectories would be representing the differences in mean time of therapy, resulting in many constant group trajectories. To a lesser degree, patients may also exhibit different levels in their attempt probability and conditional standard deviation. However, in consideration of the increased model complexity with an increasing number of latent classes, we opt for simplifying the class model. We therefore only include a random intercept $\gamma _{i}^{(g)}\sim N(0,\sigma _{\gamma })$ for the mean level. Each latent class is described by a model, where the model for class *g* is described by

8 $$\begin{array}{*{20}l} \mu_{i,j}^{(g)} & =\eta_{1,i,j}^{(g)}=\beta_{1,0}^{(g)}+\beta_{1,1}^{(g)}t_{i,j}+\beta_{1,2}^{(g)}t_{i,j}^{2}+\gamma_{i}^{(g)}, \\ \log\sigma_{i,j}^{(g)} & =\eta_{2,i,j}^{(g)}=\beta_{2,0}^{(g)}+\beta_{2,1}^{(g)}t_{i,j}+\beta_{2,2}^{(g)}t_{i,j}^{2}, \\ \log\frac{\nu_{i,j}^{(g)}}{1-\nu_{i,j}^{(g)}} & =\eta_{3,i,j}^{(g)}=\beta_{3,0}^{(g)}+\beta_{3,1}^{(g)}t_{i,j}+\beta_{3,2}^{(g)}t_{i,j}^{2}. \end{array} $$

Each of these class models represents a proportion of the overall heterogeneity in the data. The overall model is given by

9 $$ f(y_{i,j};\boldsymbol{\Theta},\boldsymbol{\pi})=\sum_{g=1}^{G}\pi_{g}f_{g}\left(y_{i,j};\boldsymbol{\beta}_{1}^{(g)},\boldsymbol{\beta}_{2}^{(g)},\boldsymbol{\beta}_{3}^{(g)},\sigma_{\gamma}\right)  $$

where ***Θ***={***θ***^(1)^,…,***θ***^(*G*)^} comprises the group model parameters, *f*_*g*_ denotes the model for group *g*, and ***π*** is the vector of group proportions *π*_*g*_ for group *g* with *π*_*g*_≥0 and $\sum _{g}\pi _{g}=1$. The class assignment of patients is probabilistic, which is in contrast to other approaches such as longitudinal *k*-means (KML) where the cluster edges are well-defined but arbitrarily selected due to the distance measure used [33].

A few studies have used a similar approach in the context of hurdle modeling. Maruotti [34] proposed a longitudinal latent-class hurdle mixed effects model that accounts for missing data patterns arising from drop-outs. They applied the model for the analysis of skin cancer counts, of which the data had a considerable number of missing measurements, in addition to zero inflation. Moreover, Ma et. al [35] used a log-normal hurdle mixture to identify patterns of factors contributing to vehicle crash rates. To the best of our knowledge no studies have used a hurdle approach with within-class heterogeneity using GAMLSS up to now, in particular when combined with class-specific temporal heteroskedasticity.

### Model estimation

The analysis is performed in R 3.5.0 [36] using version 5.1-2 of the *gamlss* package [14] for the implementation of GAMLSS. The GAMLSS model is fitted using the RS algorithm proposed by Rigby & Stasinopoulos [37]. The algorithm maximizes the (penalized) maximum likelihood of the full model using expectation maximization (EM). The estimation of the random patient factor is based on penalized quasi-likelihood. The zero-truncated normal distribution is available in the *gamlss.tr* package (version 5.1-0) [38], and was adapted to account for excess zeros by the parameter *ν*.

We estimate the mixture model specified in Equation 9 using a nonparametric maximum likelihood (NPML) approach [39, 40], as implemented in the gamlssNP function in the package *gamlss.mx* (version 4.3-5) [41]. This approach describes the data heterogeneity through a non-parametric density function comprising a finite mixture [40–42]. The marginal likelihood for the data is given by

10 $$ f(\boldsymbol{y};\boldsymbol{\Theta},\boldsymbol{\pi})=\prod_{i\in I}\sum_{g=1}^{G}\left[\pi_{g}\prod_{j=1}^{J_{i}}f_{g}\left(y_{i,j};\boldsymbol{\theta}^{(g)}\right)\right].  $$

Here, the group parameters ***θ***^(*g*)^ represent the mass points of the non-parametric density, occurring with probability (i.e., the masses) *π*_1_,…,*π*_*G*_, respectively.

Each trajectory is assumed to have been generated by one of the group models, however, the true group membership is unknown. The membership of the trajectory ***y***_*i*_ to group *g* is indicated by *δ*_*i,g*_, with *δ*_*i,g*_=1 if the trajectory belongs to group *g*, and *δ*_*i,g*_=0 otherwise. The vector of group indicators for the trajectory *i* is denoted by ***δ***_*i*_=(*δ*_*i*,1_,…,*δ*_*i,G*_). We denote the set of all indicator vectors across patients by ***δ***. With this, the likelihood of the model with specified group memberships ***δ***, referred to as the complete model, is given by

11 $$\begin{array}{*{20}l} L_{c} & =L(\boldsymbol{y},\boldsymbol{\Theta},\boldsymbol{\pi},\boldsymbol{\delta})=f(\boldsymbol{y},\boldsymbol{\delta})\\ & =f(\boldsymbol{y}|\boldsymbol{\delta})f(\boldsymbol{\delta}) \\ & =\prod_{i\in I}f(\boldsymbol{y}_{i}|\boldsymbol{\delta}_{i})f(\boldsymbol{\delta}_{i}) \\ & =\prod_{i\in I}\prod_{g=1}^{G}\left[\pi_{g}^{\delta_{i,g}}\prod_{j=1}^{J_{i}}f_{g}\left(y_{i,j}\right)^{\delta_{i,g}}\right]. \end{array} $$

Here, the parameters ***Θ*** and ***π*** were left out for conciseness. A more detailed derivation is provided by Stasinopoulos et al. [41]. The log-likelihood of the complete model is given by

12 $$ \ell_{c}=\sum_{i\in I}\sum_{g=1}^{G}\delta_{i,g}\log\pi_{g}+\sum_{i\in I}\sum_{g=1}^{G}\sum_{j=1}^{J_{i}}\delta_{i,g}f_{g}(y_{i,j}).  $$

The complete model with *G* classes is equivalent to a weighted regression model over repeated data observations for *g*=1,...,*G* with an additional covariate indicating the class membership. The observations are weighted by the posterior probability

13 $$ w_{i,g}=\hat{\pi}_{i,g}=\frac{\pi_{g}\prod_{j=1}^{J_{i}}f_{g}\left(y_{i,j};\boldsymbol{\theta}^{(g)}\right)}{\sum_{g^{\prime}=1}^{G}\pi_{g^{\prime}}\prod_{j=1}^{J_{i}}f_{g^{\prime}}\left(y_{i,j};\boldsymbol{\theta}^{(g^{\prime})}\right)}.  $$

The latent class proportions of the mixture model are computed from the respective average posterior probability, given by

14 $$ \hat{\pi}_{g}=\frac{1}{|I|}\sum_{i\in I}w_{i,g}.  $$

The EM algorithm is initialized by fitting a fixed-effects GAMLSS model. In the E-step, the patient weights are updated, followed by the maximization of the weighted GAMLSS model likelihood in the M-step. The optimization process is halted when the reduction in the deviance, computed as *D*=−2 log*L*, falls below a certain threshold. In the analysis, we use a lenient threshold of 0.3, which we determined to provide sufficiently stable results due to the large amount of data, while halting relatively quickly. Details on the algorithm and the initialization are given by Einbeck & Hinde [40]. Moreover, we observed stable solutions across repeated random starts, which is in agreement with findings by other researchers [39, 40]. Nevertheless, the model does fail to converge sometimes so repeated random starts are recommended.

### Evaluation

Prior to the mixture modeling analysis, we explore several mixed models based on Equation 7, with polynomial random effects $\sum _{p=0}^{P}\gamma _{k,p,i}t_{i,j}^{p}$ of order *P* in the predictor *η*_*k*_ of the respective distributional parameter. In addition we estimate a fixed effects model as a baseline.

We assess the model fit of the models by investigating the standardized residuals for normality, using a detrended quantile-quantile (Q-Q) plot. The different models are compared using the Akaike information criterion (AIC). The AIC measures the amount of information lost about the data by the model representation while penalizing overfitting. It is defined as AIC=2*m*−2 log*L*, where *m* is the number of model parameters, and *L* is the likelihood of the model. This is a specific case of the generalized Akaike information criterion (GAIC) [43], which is recommended for comparing non-nested models [14]. Only models that converged successfully are evaluated. Likelihood ratio tests were considered but yielded consistent p-values of zero for any improvement in AIC due to the large sample size, and therefore we do not report them in the results section. Lastly, we measure the separation between classes in terms of the relative entropy [44], given by

15 $$ \mathrm{relative\:entropy}=1-\frac{\sum\sum_{g=1}^{G}-\hat{\pi}_{i}^{(g)}\log\hat{\pi}_{i}^{(g)}}{|I|\log G}.  $$

For the selected mixture model we compare the subgroups in order to create distinct descriptors of the groups, and to highlight meaningful differences between the groups in terms of adherence. We assess the group trajectories on each of the distributional parameters visually. Furthermore, we explore whether the groups differ on any of the other available covariates of interest, which are the residual AHI, leakage, pressure settings, and motivation score. Here, each patient is assigned to the most likely group (i.e., modal assignment).

Although the additional covariates could have been included in the GAMLSS mixture model, this was omitted for practical reasons because the computation time would increase considerably. Moreover, preliminary tests on a random subset of 1,000 patients yielded mostly the same groups as the mixture model without the inclusion of an additional covariate.

We therefore apply a three-step approach, where in the first step the mixture model is estimated (i.e., the measurement model). In the second step, each patient is assigned to their most likely group. Lastly, the patient groups are compared on the covariates of interest. The means are compared using ANOVA F-tests, whereas the medians of skewed distributions are compared using the Kruskal-Wallis test. Due to the large sample size of this study, even small differences between groups are statistically significant. Instead we will only highlight practically significant differences that are deemed clinically relevant.

The three-step approach has been shown to lead to biased estimates on the effects of external variables [16]. We therefore considered the modified Bolck-Croon-Hagenaars (BCH) approach, which applies a correction for the misclassification errors [16, 45]. However, when applied to the case study at hand, we observed that the correction did not result in a meaningful difference in the mean estimates between groups, nor different conclusions on statistical significance. This is likely attributable to the large sample size, and low misclassification error due to the large number of observations per trajectory.

## Results

**Fig. 2:**
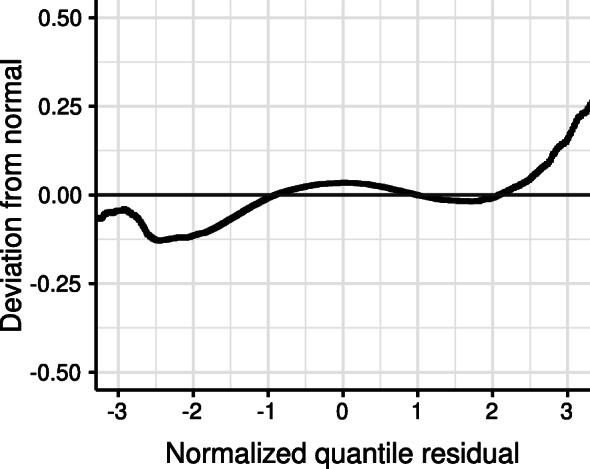
Detrended Q-Q plot of the single-group model with linear random effects

The model fits for different degrees of polynomials (*P*=0,1,2), and the fixed effects model are reported in Table 1 in terms of the AIC. An increasing order of the random effects is associated with an improved model fit. However, the model involving the quadratic random effects failed to converge despite repeated random starts. The detrended Q-Q plot of the linear random effects model shown in Fig. 2 indicates that the residual deviations from the normal distribution are closely concentrated around zero, suggesting that the normalized quantile residuals are approximately normally distributed. However, the pattern of negative deviation at the tails indicates the presence of many outliers, i.e., heavy tails.

**Table 1:** The single-group model estimates

Random effects in *η*_*k*_	AIC
None	4,070,897
Constant	3,267,326
Linear	3,209,083
Quadratic	Did not converge

### Number of groups

We determine the best fitting mixture model for 1 to 10 classes. For each number of groups, ten models were fitted using random starts, out of which the model with the lowest AIC was selected. Overall, the solutions among the repeated random starts, in the cases where convergence was reached, are stable. This is consistent with observations by Aitkin [46] for this type of estimation.

**Fig. 3:**
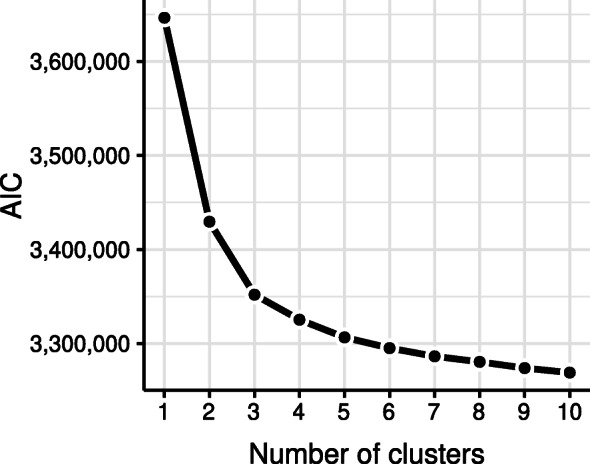
AIC for the model solutions for 1 to 10 groups

The AIC of the best model for each number of groups is shown in Fig. 3. The monotonically decreasing curve suggests a consistent but diminishing improvement in model fit with an increased number of groups. With the aim of exploring the various ways in which patients adhere to the therapy, and in consideration of the sufficient amount of data for a post-hoc analysis, a solution involving many groups is justified, and supported by the AIC and likelihood ratio test (*p*≪0.01). An alternative solution of interest would be the solution involving three groups, which is the solution at which the improvements in the consecutive models start to diminish.

We choose the nine-groups solution because it provides a better fit than solutions involving fewer groups, as indicated by the AIC. Moreover, the eight-groups solution lacks the group trajectory exhibiting considerably increase in usage over time which is present in the solution with nine groups. On the other hand, the ten-group solution is almost identical to the preferred solution, with the exception of an additional constant group trajectory which we deem to be not of interest.

**Fig. 4:**
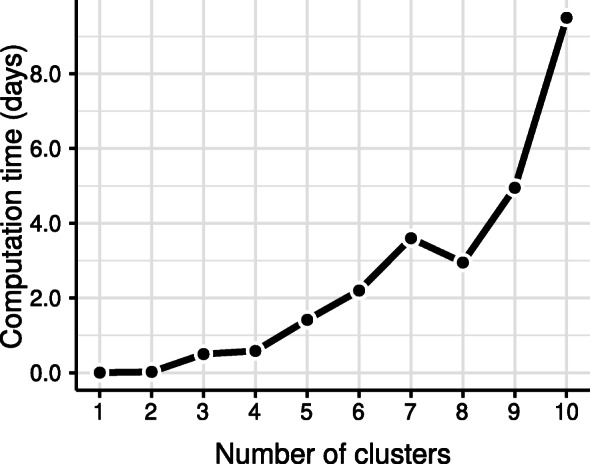
The average computation time (in days) of the mixture models for each of the different number of groups

The computation time increased considerably with an increasing number of groups, up to the point where model estimation is no longer practical. Whereas the single-group model takes 34 minutes to compute on an Intel Xeon E5-2660 (2.6 GHz) processor, the five-groups model needed 34 hours on average, and the ten-group models completed only after 228 hours on average. The average computation time for each number of groups is shown in Fig. 4. The models involving nine groups or less either converged within 50 outer iterations, or failed to convergence. The ten-groups models converged within 78 iterations.

### Adherence groups

**Fig. 5:**
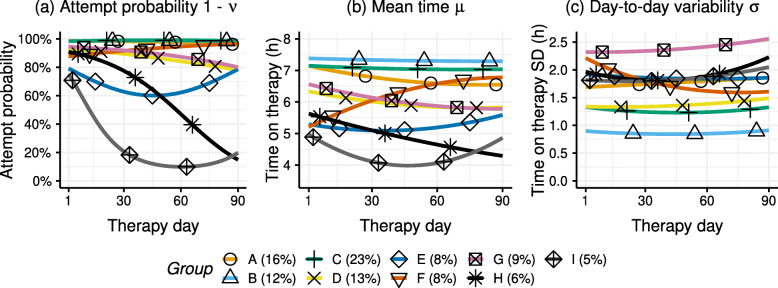
The identified group trajectories for each distributional parameter

The nine group trajectories are shown in Fig. 5 for each of the distributional parameters, with the model coefficients shown in Table 2. The means of the group trajectories are reported for day 1, 45 and 90 in Table 3.

**Table 2:** Group trajectory coefficients

	Group	$\hat {\pi }_{g}$	logit *ν*			*μ*			log*σ*		
			*β*_2,0_	*β*_2,1_	*β*_2,2_·10^3^	*β*_1,0_	*β*_1,1_	*β*_1,2_·10^3^	*β*_2,0_	*β*_2,1_·10^2^	*β*_2,2_·10^4^
A	Variable users	16%	-4.2	.0032	.093	7.1	-.015	.093	.52	.10	.0057
B	Consistent users	12%	-4.1	-.015	.18	7.4	-.0021	.012	-.11	-.31	.36
C	Good users	23%	-4.1	-.025	.26	7.1	-.0034	.023	.29	-.36	.39
D	Stable decliners	13%	-2.6	.018	-.031	6.3	-.014	.090	.29	-.064	.20
E	Strugglers	8%	-1.3	.042	-.46	5.3	-.012	.17	.65	-.15	.12
F	Improvers	8%	-2.0	-.015	-.014	5.2	.035	-.19	.79	-.88	.57
G	Variable decliners	9%	-2.8	.014	.029	6.6	-.018	.096	.84	-.011	.13
H	Dropouts	6%	-2.3	.032	.15	5.6	-.020	.053	.68	-.49	.69
I	Early dropouts	5%	-1.1	.11	-.94	5.0	-.044	.47	.60	-.13	.29

**Table 3:** The group trajectories at day 1, 45 and 90

	Group	Attempt probability 1−*ν*			Mean time *μ* (h)			SD time *σ* (h)		
		Day 1	Day 45	Day 90	Day 1	Day 45	Day 90	Day 1	Day 45	Day 90
A	Variable users	99%	98%	96%	7.1	6.7	6.5	1.7	1.8	1.9
B	Consistent users	98%	99%	98%	7.4	7.3	7.3	.90	.84	.90
C	Good users	98%	99%	99%	7.1	7.0	7.0	1.3	1.2	1.3
D	Stable decliners	93%	87%	78%	6.3	5.9	5.8	1.3	1.4	1.5
E	Strugglers	79%	59%	77%	5.3	5.1	5.6	1.9	1.8	1.9
F	Improvers	88%	93%	97%	5.2	6.4	6.8	2.2	1.7	1.6
G	Variable decliners	95%	90%	80%	6.6	6.0	5.8	2.3	2.4	2.6
H	Dropouts	91%	65%	15%	5.6	4.9	4.3	2.0	1.8	2.2
I	Early dropouts	75%	11%	17%	5.0	3.9	4.8	1.8	1.8	2.0

The value range of the group trajectories for the mean time on therapy is surprisingly narrow, with only a 3.5 hour difference between the lowest and highest group. The small proportion of patients that fall outside of this range are accounted for by the random intercept in the group models. The difference between the mean group trajectories is especially small in relation to the day-to-day standard deviation of usage, with standard deviations ranging between 0.84 and 2.4 h. In addition, there is a considerable spread within groups on the mean intercept, with *σ*_*γ*_=1.5 hours. Despite of the high day-to-day variability, the large number of observations available per patient allows for a reliable classification, as indicated by the high relative entropy of 0.93.

The group trajectories show a gradual change in mean usage over time, which is possibly due to patients changing their usage at different moments throughout the therapy. In contrast, the changes in attempt probability are more profound, with a significant group of patients that tend to nearly cease the therapy within the first month. Overall, the attempt probability and its change over time differ considerably between groups, with some groups achieving near-perfect consistency in daily attempts (99%), and other groups using the therapy sporadically (attempts on 15% of days) towards day 90. Several of the groups exhibit a small increase in usage variability over time. In some groups, this change in variability appears to coincide with a change in mean usage, possibly indicating a mean-variance relationship. In general, the group trajectories with higher usage have lower variability.

Group A, B, and C represent highly consistent users, making up the majority of patients (51%). Group B (12%) and C (23%) represent patients that have no trouble adhering to therapy, with a consistent average attempt probability of 99%, usage averaging around 7 h, and having the lowest day-to-day variability of all groups. The discerning factor between the groups is the day-to-day variability of group C of 1.3 h, compared to the even lower standard deviation of 0.90 hours for group B.

The patients of group A (16%) achieve nearly the same consistency in attempts as group B and C, but show a decrease in usage of half an hour throughout the therapy. Moreover, the standard deviation is considerably higher at 1.8 h.

In terms of usage, group D (13%) and G (9%) follow a trajectory similar to group A, but have a reduced number of attempts by day 90 from 94% to 80%. The difference between these two groups lies in their day-to-day variability. With a standard deviation of around 2.5 hours, patients in group G have the highest variability of all groups by far.

Group E, F, H, and I represent struggling patients (for a total of 27% of patients, with 8%, 8%, 6% and 5% respectively). These patients tend to have a lower average usage already at the start of therapy. Whereas the patients from group F improve with time, the usage of the other groups either remains constant or decreases over time. The strugglers in group E exhibit a stable usage over time, but a diminished number of attempts around the second month of therapy. Group H and I comprise patients who decrease in number of attempts, the separating factor between the groups is the time at which attempts are no longer made or only occur sporadically.

**Fig. 6:**
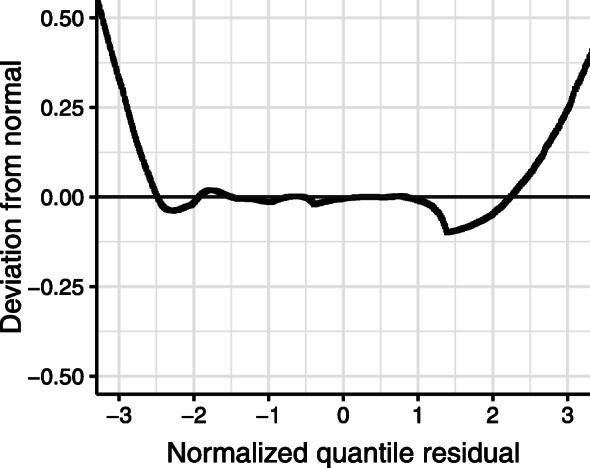
Detrended Q-Q plot of the preferred mixture solution

We assess the fit of the mixture model to the data using the detrended Q-Q plot shown in Fig. 6. The normalized quantile residuals closely follow a normal distribution, with the exception of the heavy tails, indicating that the data contains considerable outliers with respect to expected group trajectory. The deviation around 1.3 can primarily be attributed to observations from patients of the early dropouts group I, suggesting that more group trajectories are needed to adequately describe the trajectories of these patients, or that a different model is needed for this specific group.

### Group comparison

We compare the identified groups on the covariates described in the “Data” section based on the measurements in the first week, and across all 90 days of therapy. The group mean and median values for each covariate, along with the standard deviation and interquartile range respectively, are reported in Table 4. The median attempted days, time on therapy and day-to-day variability are reported for reference. The proportion of compliant days was determined by the number of days with time on therapy exceeding 4 hours out of 90 days. The pressure-related covariates were only available in 675 patients. Moreover, the minimum and maximum CPAP pressure settings remained unchanged in 81% of patients, therefore the median values in week 1 are not reported.

**Table 4:** Unadjusted group estimates for the covariates (between-patient standard deviation enclosed in parentheses). The median is reported for covariates with a considerably skewed distribution (lower and upper quartiles enclosed in brackets)

	Variable users (16%)	Consistent users (12%)	Good users (23%)	Stable decliners (13%)	Strugglers (8%)	Improvers (8%)	Variable decliners (9%)	Dropouts (6%)	Early drop-outs (5%)
	A	B	C	D	E	F	G	H	I
Attempted days (%)	$\begin {array}{l} 98\\[-2.5pt] [97,100] \end {array}$	$\begin {array}{l} 100\\[-2.5pt] [98,100] \end {array}$	$\begin {array}{l} 100\\[-2.5pt] [98,100] \end {array}$	$\begin {array}{l} 88\\[-2.5pt] [82,91] \end {array}$	$\begin {array}{l} 69\\[-2.5pt] [60,74] \end {array}$	$\begin {array}{l} 93\\[-2.5pt] [90,97] \end {array}$	$\begin {array}{l} 89\\[-2.5pt] [84,93] \end {array}$	$\begin {array}{l} 59\\[-2.5pt] [50,68] \end {array}$	$\begin {array}{l} 26\\[-2.5pt] [14,33] \end {array}$
Time on therapy (h)	6.7 (1.3)	7.3 (.91)	7.1 (1.1)	6.0 (1.2)	5.3 (1.3)	6.3 (1.3)	6.1 (1.4)	5.1 (1.5)	4.7 (1.9)
Time on therapy SD (h)	1.8 (.20)	.86 (.13)	1.3 (.14)	1.4 (.23)	1.8 (.39)	1.8 (.27)	2.3 (.40)	1.8 (.46)	1.6 (.56)
Compliant days (%)									
Week 1	$\begin {array}{l} 100\\[-2.5pt] [86,100] \end {array}$	$\begin {array}{l} 100\\[-2.5pt] [100,100] \end {array}$	$\begin {array}{l} 100\\[-2.5pt] [100,100] \end {array}$	$\begin {array}{l} 86\\[-2.5pt] [71,100] \end {array}$	$\begin {array}{l} 57\\[-2.5pt] [43,86] \end {array}$	$\begin {array}{l} 71\\[-2.5pt] [43,86] \end {array}$	$\begin {array}{l} 86\\[-2.5pt] [71,100] \end {array}$	$\begin {array}{l} 71\\[-2.5pt] [57,86] \end {array}$	$\begin {array}{l} 43\\[-2.5pt] [14,71] \end {array}$
All	$\begin {array}{l} 90\\[-2.5pt] [83,96] \end {array}$	$\begin {array}{l} 99\\[-2.5pt] [98,100] \end {array}$	$\begin {array}{l} 98\\[-2.5pt] [94,99] \end {array}$	$\begin {array}{l} 79\\[-2.5pt] [69,86] \end {array}$	$\begin {array}{l} 50\\[-2.5pt] [36,61] \end {array}$	$\begin {array}{l} 81\\[-2.5pt] [71,89] \end {array}$	$\begin {array}{l} 70\\[-2.5pt] [60,80] \end {array}$	$\begin {array}{l} 41\\[-2.5pt] [29,53] \end {array}$	$\begin {array}{l} 11\\[-2.5pt] [4.4,22] \end {array}$
Residual AHI (events/h)									
Week 1	$\begin {array}{l} 2.7\\[-2.5pt] [1.5,4.8] \end {array}$	$\begin {array}{l} 2.5\\[-2.5pt] [1.3,4.7] \end {array}$	$\begin {array}{l} 2.4\\[-2.5pt] [1.2,4.6] \end {array}$	$\begin {array}{l} 2.9\\[-2.5pt] [1.5,5.3] \end {array}$	$\begin {array}{l} 2.9\\[-2.5pt] [1.5,5.8] \end {array}$	$\begin {array}{l} 3.1\\[-2.5pt] [1.5,5.5] \end {array}$	$\begin {array}{l} 3.0\\[-2.5pt] [1.7,5.3] \end {array}$	$\begin {array}{l} 3.1\\[-2.5pt] [1.7,5.9] \end {array}$	$\begin {array}{l} 2.6\\[-2.5pt] [1.3,5.3] \end {array}$
All	$\begin {array}{l} 2.3\\[-2.5pt] [1.3,4.0] \end {array}$	$\begin {array}{l} 2.1\\[-2.5pt] [1.3,3.9] \end {array}$	$\begin {array}{l} 2.1\\[-2.5pt] [1.1,3.7] \end {array}$	$\begin {array}{l} 2.5\\[-2.5pt] [1.4,4.3] \end {array}$	$\begin {array}{l} 2.8\\[-2.5pt] [1.5,5.2] \end {array}$	$\begin {array}{l} 2.4\\[-2.5pt] [1.3,4.2] \end {array}$	$\begin {array}{l} 2.7\\[-2.5pt] [1.5,4.4] \end {array}$	$\begin {array}{l} 2.8\\[-2.5pt] [1.6,5.1] \end {array}$	$\begin {array}{l} 2.6\\[-2.5pt] [1.4,4.9] \end {array}$
High residual AHI (%)									
Week 1	3.4	3.1	3.0	3.6	4.7	4.9	4.3	4.6	5.1
All	2.1	1.9	1.6	2.4	4.0	2.2	2.6	3.6	4.5
Leakage (L/min)									
Week 1	38 (13)	37 (12)	38 (13)	37 (13)	36 (13)	38 (14)	36 (12)	36 (12)	36 (13)
All	38 (12)	37 (11)	38 (12)	37 (13)	37 (12)	38 (12)	37 (12)	36 (12)	37 (13)
SD leakage (L/min)									
Week 1	$\begin {array}{l} 4.0\\[-2.5pt] [2.4,6.9] \end {array}$	$\begin {array}{l} 3.5\\[-2.5pt] [2.0,5.9] \end {array}$	$\begin {array}{l} 3.8\\[-2.5pt] [2.2,6.5] \end {array}$	$\begin {array}{l} 3.7\\[-2.5pt] [2.1,6.9] \end {array}$	$\begin {array}{l} 4.0\\[-2.5pt] [2.1,7.5] \end {array}$	$\begin {array}{l} 4.4\\[-2.5pt] [2.3,8.0] \end {array}$	$\begin {array}{l} 3.9\\[-2.5pt] [2.1,6.7] \end {array}$	$\begin {array}{l} 4.0\\[-2.5pt] [2.2,7.0] \end {array}$	$\begin {array}{l} 3.8\\[-2.5pt] [2.2,8.2] \end {array}$
All	$\begin {array}{l} 4.7\\[-2.5pt] [3.1,7.2] \end {array}$	$\begin {array}{l} 3.6\\[-2.5pt] [2.4,5.3] \end {array}$	$\begin {array}{l} 4.1\\[-2.5pt] [2.7,6.2] \end {array}$	$\begin {array}{l} 4.6\\[-2.5pt] [2.8,7.4] \end {array}$	$\begin {array}{l} 5.0\\[-2.5pt] [3.1,8.1] \end {array}$	$\begin {array}{l} 5.0\\[-2.5pt] [3.0,7.7] \end {array}$	$\begin {array}{l} 4.9\\[-2.5pt] [3.2,7.4] \end {array}$	$\begin {array}{l} 5.1\\[-2.5pt] [3.0,8.7] \end {array}$	$\begin {array}{l} 5.2\\[-2.5pt] [3.3,9.1] \end {array}$
Min pressure (cmH _2_O)	$\begin {array}{l} 6\\[-2.5pt] [5,8] \end {array}$	$\begin {array}{l} 6\\[-2.5pt] [5,8] \end {array}$	$\begin {array}{l} 6\\[-2.5pt] [5,8] \end {array}$	$\begin {array}{l} 6\\[-2.5pt] [5,8] \end {array}$	$\begin {array}{l} 6\\[-2.5pt] [4,7] \end {array}$	$\begin {array}{l} 6\\[-2.5pt] [5,8] \end {array}$	$\begin {array}{l} 6.0\\[-2.5pt] [5.0,8.0] \end {array}$	$\begin {array}{l} 5.0\\[-2.5pt] [4.0,6.0] \end {array}$	$\begin {array}{l} 5.0\\[-2.5pt] [4.0,7.0] \end {array}$
Avg pressure (cmH _2_O)									
Week 1	9.4 (3.1)	9.3 (3.2)	9.0 (2.6)	8.4 (2.0)	9.6 (2.9)	9.1 (3.3)	8.3 (2.5)	8.5 (2.4)	9.4 (2.8)
All	9.6 (3.1)	9.3 (3.0)	9.3 (2.9)	8.7 (2.3)	9.5 (3.1)	9.2 (2.9)	8.6 (2.9)	8.8 (2.5)	9.2 (2.9)
Max pressure (cmH _2_O)	$\begin {array}{l} 15\\[-2.5pt] [12,20] \end {array}$	$\begin {array}{l} 15\\[-2.5pt] [13,16] \end {array}$	$\begin {array}{l} 15\\[-2.5pt] [12,20] \end {array}$	$\begin {array}{l} 15\\[-2.5pt] [12,20] \end {array}$	$\begin {array}{l} 13\\[-2.5pt] [11,16] \end {array}$	$\begin {array}{l} 15\\[-2.5pt] [13,20] \end {array}$	$\begin {array}{l} 15\\[-2.5pt] [12,20] \end {array}$	$\begin {array}{l} 15\\[-2.5pt] [11,16] \end {array}$	$\begin {array}{l} 15\\[-2.5pt] [12,20] \end {array}$
Patient motivation (%)									
Week 1: score<4	1.7	1.1	.67	2.4	3.0	1.6	1.5	5.6	6.2
Week 1: 4≤score≤6	9.2	4.8	5.9	12	17	12	9.6	17	19
Week 1: score>6	89	94	93	86	80	86	89	77	74

Already in the first week of therapy, group A, B and C comprise relatively more compliant patients than the other groups, with the majority of patients achieving daily compliance. This is in contrast to group E and I with a respective proportion of only 57% and 43%. All groups except F (the improvers) show a decline in compliant days over time. The decline is considerable for the drop-out groups H and I, which can be attributed to the reduced number of attempts over time.

With respect to the residual AHI, the median and lower percentiles between groups is minute. In contrast, the differences at the 75^th^ percentile and higher are more pronounced, where the AHI of groups E, H and I is higher by 1 event/hour. It is worth noting that only the early drop-outs group (I) do not show a decrease in AHI relative to the first week. As patients with consistently high AHI may have abandoned the therapy prematurely, we also investigate the average proportion of patient residual AHI measurements exceeding 15 events/hour across the groups (referred to as high residual AHI in Table 4). Differences between the groups are present from week 1 onward, notably between the more adherent groups (A-D) and the other groups. Even more so, the differences are greater in the period following the first week, with the groups exhibiting struggling or drop-out behavior (groups E, H, I) having over twice the rate of high residual AHI compared to the adherent groups A, B and C.

Leakage was found to be practically identical between groups. We therefore also investigated cases of high leakage. For the high leak analysis, the available research data did not allow for an adjustment of the relevant factors for leakage (most importantly, the type of mask used). Instead, we therefore evaluated the within-patient leakage variability. We computed the standard deviation of the day-to-day differences in leakage for each patient (referred to as SD leakage in Table 4). Leakage variability was found to be highest in the drop-out groups (H and I), and lowest in the consistent users group (B).

The drop-out groups (H and I) tend to have a higher proportion of patients with the lowest possible minimum CPAP pressure of 5 cm*H*_2_O compared to the other groups. This could be due to these groups comprising patients with a less severe form of sleep apnea, or a suboptimal device configuration.

The motivation score provided by patients during the first week of therapy ranges from 1 to 10. There are considerable proportional differences between groups. The drop-out and struggling groups have a higher proportion of patients who rated their motivation below 4. Conversely, patients in groups with the highest level of adherence, B and C, patients were more likely to be motivated from the start.

## Discussion

Previous studies have explored CPAP adherence patterns using relatively small datasets involving fewer than 250 patients. Moreover, the behavioral characteristics on which the clusters in these studies are based are more limited in scope, with most studies using the time on therapy as the response to cluster on [12, 13]. Although the number of clusters that could be found in these studies was largely limited by sample size (as pointed out by Wohlgemuth et al. [10]), the fewer model characteristics and lower granularity of measurements have likely also played a role. Despite the different selection of patients, characteristics, and therapy duration, some agreements can be found with other studies on the mean usage over time. In particular, constant and declining patterns of usage are commonly found.

The proposed latent-class hurdle model based on GAMLSS allows for a more detailed description of adherence over time in OSA patients undergoing CPAP therapy, modeling changes in attempts, time on therapy, and day-to-day variability in a single model. The nine identified group trajectories emphasize the complexity surrounding CPAP therapy adherence. A narrow majority of patients who used the DreamMapper application (51%) exhibited a stable adherence pattern across the first 90 days of therapy, with the most distinguishing characteristic being the day-to-day variability. Other patients exhibit a change over time; typically a decline. The group trajectories involving a change over time have similar characteristics in the first week of therapy, suggesting that there are other factors involved that determine how the patient adherence shifts over time. Identifying these contributing factors presents opportunities for early interventions [47].

We have identified several possible contributing factors. The largest differences were observed in the motivation score. This score, assessed in the first week of therapy, showed large proportional differences, where patients with low motivation are more likely to belong to the drop-out groups. Including additional psychosocial factors studied in literature would likely help to explain the observed group trajectories further [48, 49]. The comparison of residual AHI yielded only minor differences in median AHI between groups. However, the differences were more pronounced in the upper quartile, indicating that struggling and drop-out groups may comprise a subgroup of patients with a higher residual AHI. This is further demonstrated by the different occurrence rates of high residual AHI, with the strugglers and drop-out groups having over twice the rate compared to the most adherent groups. Similarly, variability in leakage was found to differ after the first week of therapy, with the drop-out groups having the highest variability in leakage.

It was essential to model the conditional mean usage by a random intercept because of the variability in intercept between patients with the same change over time. This variance component was not needed for the attempt probability as the patterns of the drop-out and declining groups are much more distinct from the other groups. Although the inclusion of day-to-day variability in the model resulted in the identification of additional groups, the day-to-day variability showed little change over time in the majority of patients, with the improvers from group F being the notable exception.

All group trajectories remained above the minimum compliance threshold of 4 hours, suggesting that even in the group with the lowest average time on therapy (group I, the early drop-outs), patients met the threshold on average. Our findings suggest that across groups, therapy adherence is mostly affected by a decrease in attempts over time, suggesting that the focus on encouraging patients to attempt the therapy on a daily basis is more important than increasing the hours of usage above the compliance threshold.

It is important to note that because most patients that abandon the therapy do so within 90 days, our results are biased towards the more adherent patients. We suspect this is why the groups with average usage below 4 hours identified by Babbin et al. [12], Wang et al. [13] and Wohlgemuth et al. [10] were not found in our analysis. On a similar note, our estimates of therapy factors such as AHI or leakage are also likely to be lower, as significant issues on these aspects could contribute to patients abandoning therapy. Furthermore, due to the selection of patients who used the DreamMapper application, the findings may not be representative of the general sleep apnea population [17].

The residual analysis showed that the model fits the data adequately, with only the tails of the distribution departing from a normal distribution. The heavy tails could likely be accounted for by including more random effects into the class models, allowing a greater range of patient-specific deviations from the group trajectory. Alternatively, a truncated distribution with a heavier tail than the truncated normal distribution (e.g., the t-distribution) could be used. The choice for the normal distribution was a trade-off between model complexity and model fit, as both of the proposed alternatives would increase the model complexity and estimation time considerably.

Due to the excessive computation time for the nine- and ten-class models, we restricted the regression analyses to a random subset of 10,000 patients out of the available 37,235 patients. In order to ensure that this would not affect our results, we conducted a preliminary analysis where we visually determined that the group trajectories were sufficiently stable from random samples comprising 5,000 patients each. Considering the large number of data points per patient, a feature-based approach could have possibly provided a similar solution in a significantly shorter amount of time. In such an approach, the patient trajectories are estimated independently, after which latent class analysis is performed on the trajectory coefficients.

Bearing in mind the high day-to-day variability observed within patients, the model fit could be improved further if factors can be determined that explain some of the observed variability. Moreover, the hurdle model assumes that the occurrence of intermittent days are independent events while the factors that affect attempts may last several days (e.g., illness). Modeling intermittent days as a state change lasting one or more days could provide an improved description, especially for patients who are struggling with the therapy.

Overall, the proposed methodology provides a detailed description of patient adherence behavior over time, especially in comparison to earlier studies. Our approach is useful to researchers, clinicians, and durable medical equipment (DME) providers for discovering common patterns of adherence in their (sub)population of interest, and gaining insights into how adherence behavior differs between patients. Moreover, such insights could help DME providers better identify the risk of overpay in reimbursement claims based on adherence levels. Lastly, the proposed model can be used to assign new patients to the most likely adherence pattern, enabling the detection of behaviors of interest. In particular, identifying problematic patterns of adherence may help in better recognizing and targeting patients who are struggling with the therapy.

## Conclusion

We have demonstrated the feasibility and benefits of applying a latent-class heteroskedastic hurdle trajectory model to adherence data with a large number of patients. The inclusion of the hurdle model and the heteroskedastic model into the mixture model enabled the discovery of additional adherence patterns, and a more descriptive representation of patient behavior over time. Most importantly, the analysis revealed a strong non-linear association between the progression of attempts over time, and the average time on therapy. The methodology presented here can be applied to behavioral data in other domains involving the tracking of compliance over time.

## Data Availability

The data that support the findings of this study are available from Philips Respironics but restrictions apply to the availability of these data, which were used under license for the current study, and so are not publicly available. Data are however available from the authors upon reasonable request and with permission of Philips Respironics.

## Abbreviations

AHI: Apnea-hypopnea index
CPAP: Continuous positive airway pressure
DME: Durable medical equipment
GAMLSS: Generalized additive modeling for location, scale, and shape
GMM: Growth mixture modeling
OSA: Obstructive sleep apnea

## Footnotes

1 This random subset was selected due to computational considerations in the cluster analysis for models with a large number of clusters (up to 10).
